# Walking in a self-selected enjoyable colored environment: exploring gender-specific effects and dynamics of affects, perceived exertion, heart rate, and preferred walking speed

**DOI:** 10.3389/fpsyg.2025.1502172

**Published:** 2025-01-21

**Authors:** Youmna Elsayed Hassanein, Walid Briki, Lina Majed

**Affiliations:** ^1^College of Health and Life Sciences, Hamad Bin Khalifa University, Doha, Qatar; ^2^Sport Science Program, College of Arts and Sciences, Qatar University, Doha, Qatar; ^3^Independent Researcher, Grasse, France

**Keywords:** color psychology, approach motivation, mood, physiology, motor behavior, sex, wellbeing

## Abstract

**Aim:**

Existing literature suggests that exposure to colored environments can influence psychological, physiological, and behavioral responses. This study examined the effects of experiencing a self-selected enjoyable colored environment, compared to a neutral one, on psychological and physiological responses, as well as preferred walking speed during a 20-min treadmill walk.

**Methods:**

Eighty participants (40 females) were randomly assigned to either an experimental group (preferred colored environment) or a control group (neutral gray environment). Data were collected at five regular intervals during the treadmill walk.

**Results:**

Participants walked significantly slower in the colored environment compared to the neutral one, with this effect more pronounced in females, who significantly reduced their walking speed, while males remained unaffected. The walking task generally decreased depression and confusion levels; females showed a stronger reduction in depression, while males exhibited no significant change. During the task, females revealed significantly higher levels of perceived exertion and heart rate compared to males. A dynamic pattern emerged over time, with increases in perceived exertion, arousal, heart rate, and walking speed, while pleasure levels remained stable.

**Conclusions:**

The findings suggest that females are more responsive—psychologically, physiologically, and behaviorally—to both the preferred colored environment and the walking task, demonstrating a greater tendency to derive wellbeing benefits from subtle stimuli. Furthermore, the observed dynamic pattern suggests the view that self-paced walking is intrinsically governed by a pleasure-driven dynamic. These insights offer valuable implications for designing tailored interventions and strategies to promote physical activity through walking. Future research should further explore the role of color preference perception and self-paced walking in enhancing wellbeing, particularly within exercise and rehabilitation contexts.

## 1 Introduction

A growing body of literature is focusing on the psychological and physiological properties of colors and their impact on performance and behaviors. Numerous findings highlighted the influence of perceiving colors, particularly green, blue, and red on people's cognitions, emotions, physiological responses, motor behaviors, and performance (Akers et al., 2012; Barton et al., 2012; Elliot and Maier, 2014; Ilie et al., 2008; Wilms and Oberfeld, 2018). Colors can be objectively characterized by using the common hue, saturation, and value (HSV) model indicating the wavelength, chroma or intensity, and luminosity or brightness, respectively (Ibraheem et al., 2012).

### 1.1 Colors and avoidance-approach motivation

Studies linked the color red to aggression and dominance across various animal species (Andersson et al., 2002; Waitt et al., 2003) and humans (Briki and Hue, 2016; Feltman and Elliot, 2011). These links may account for the beneficial effects of wearing red on self-confidence (Recours and Briki, 2015) and sports performance, notably in tasks requiring strength and power (Dreiskaemper et al., 2013), as well as in team and combat sports (Attrill et al., 2008). However, studies also highlighted the potentially adverse effects of the color red, showing that perceiving it could negatively affect performance-related emotions by increasing cognitive anxiety (Recours and Briki, 2015), characterized by feelings of worry about the outcome, and hinder sports performance in activities demanding sustaining efforts (Briki et al., 2015).

Similarly, exposure to red appeared to negatively affect cognitive reasoning. For instance, Rutchick et al. (2010) found that exposure to the color red prompted a stronger tendency to associate with negative concepts, such as “failure”, rather than positive ones, such as “fairness.” When completing a word starting with “fai_” (e.g., “fail” or “fair”), participants exposed to red were more likely to write “fail” than “fair.” Elliot and his colleagues showed that participants exposed to red before completing an intellectual task performed worse compared to those exposed to green or gray (e.g., Lichtenfeld et al., 2009; Maier et al., 2008). These negative effects may be attributed to *avoidance motivation* (Elliot and Maier, 2014), which can be defined as “…the energization of behavior by, or the direction of behavior away from, negative stimuli (objects, events, possibilities)” (Elliot, 2006, p. 112).

In contrast, the color green has been linked to calmness, wellbeing, and positive mood (Kaya and Epps, 2004), suggesting that humans have an innate connection to nature, as it provides essential resources for fulfilling basic needs, such as shelter, water, food, materials for tool-making, as well as opportunities for rest and safety (Wilson, 1984). In sports, Briki et al. (2015) showed that exposure to green enhanced enjoyment during endurance-based athletic efforts. Similarly, in exercise settings, exposure to green—particularly in a green environment—was shown to reduce ratings of perceived exertion (RPE), improve mood (Akers et al., 2012; Focht, 2009), and lower heart rate (HR) during walking (Briki and Majed, 2019), compared to exercising in a gray or red environment. The concept of “green exercise” (Kaplan, 2001), which refers to engaging in physical activity within natural settings, underscores the numerous benefits of exercising in such environments, including enhanced enjoyment, self-esteem, and motivation, as well as reduced anxiety (Barton et al., 2012; Kaya and Epps, 2004). However, even in indoor exercise settings, the color green alone was found to provide similar benefits (Akers et al., 2012; Briki and Majed, 2019). These positive effects may be due to *approach motivation* (Elliot and Maier, 2014), which can be defined as “…the energization of behavior by, or the direction of behavior toward, positive stimuli (objects, events, possibilities)” (Elliot, 2006, p. 112).

In physical rehabilitation contexts, it was shown that the color of the indoor environment in healthcare facilities can influence patients' physical and mental health (Dalke et al., 2006; Hettiarachchi and Perera, 2022; Salonen et al., 2013). For instance, light-colored rooms can positively impact the speed of recovery and wellbeing in patients (Eminovic et al., 2022). Moreover, green walls can significantly reduce symptoms of stress, anxiety, and depression, compared to blue or yellow (Hettiarachchi and Perera, 2022), while orange-colored environments in hospitals are associated with increased arousal (Dijkstra et al., 2008). This evidence suggests that the strategic use of environmental colors in healthcare settings can influence approach and avoidance motivations, with calming colors like green fostering approach-related states of mind, such as wellbeing, while activating colors like orange may induce avoidance-related arousal.

### 1.2 Focusing on enjoyable environmental colors

Given the evidenced benefits of environmental colors associated with approach motivation, particularly their positive effects on physical and mental health, further research is needed to deepen our understanding of the psychological, physiological, and behavioral responses to exercising within comfortable or enjoyable colored environments. To date, only one study, that of Briki and Majed (2019), explored the impact of colored environments on psychophysiological responses and self-selected behaviors, such as preferred walking speed (PWS).

Their findings showed that participants walking at their PWS in a green environment had significantly lower HR compared to those in red or white environments, suggesting that engaging in low-intensity physical activity within a green environment may promote wellbeing by eliciting a calming effect on the human organism. This is consistent with Briki and Hue's (2016) study, which identified green as a more pleasurable color. Additionally, Briki and Majed (2019) found that, regardless of the color conditions, participants' RPE, HR, and PWS increased progressively over time, which the authors attributed to the natural progression toward the ventilatory threshold—the point at which aerobic metabolism transitions to anaerobic metabolism (see Smith et al., 2015). Lastly, participants showed higher vigor scores post-walking compared to pre-walking, leading the authors to describe self-paced walking as a form of “volitional exercise” performed under peaceful conditions, potentially accounting for its positive effects on mood states.

Moreover, the authors emphasized the importance of considering gender-specific interpretations of colors in future research, suggesting that the effects of colors on the human organism may result from the interplay of innate and cultural factors. Building on their perspective, we can suggest that males and females may not only display different responses to identical color stimuli but may also choose distinct colors when instructed to identify, for example, their “most enjoyable color.”

The literature evidenced varying effects of colors influenced by factors such as culture (Kocher and Sutter, 2008), ethnicity (Lee and Lee, 2021), and gender (Ioan et al., 2007). Cultural influences of colors on performance can be inferred from conflicting results, such as the enhancement of performance by the color red in the English Premier League (Attrill et al., 2008), which was not evidenced in football leagues from other countries, such as Poland, Spain, or Germany (García-Rubio et al., 2011; Kocher and Sutter, 2008; Szmajke and Sorokowski, 2006). These differences are thought to stem from culturally learned experiences that may amplify or weaken the effect of a specific color (Kocher and Sutter, 2008). In Italy, a study reported that blue placebo sleeping pills had stronger effects on women compared to men, who may associate blue with stimulation and excitement due to its connection with the national football team (Cattaneo et al., 1970; Moerman, 2002).

### 1.3 The present research

Building on Briki and Majed's (2019) findings and insights, the present study pursued four distinct objectives. First, it investigated the effects of individually preferred environmental colors, focusing on how one's most enjoyable colored environment influences psychological (i.e., RPE, pleasure, arousal), physiological (i.e., HR), and behavioral (i.e., PWS) parameters during a 20-min walking session. Second, it examined how the simple fact of self-paced walking could impact such responses over time. Third, it explored whether walking at a self-selected pace could promote a more positive mood. Finally, it investigated potential gender differences across all these responses.

Based on the authors' findings, we formulated the following hypotheses:

(a) Walking in a self-selected, enjoyable colored environment was expected to result in a significant reduction in HR, compared to walking in a neutral environment.
(b) Regardless of the color conditions, RPE, HR, and PWS were expected to increase progressively over time.
(c) Pre- and post-task mood comparisons were anticipated to reveal an increase in vigor following the walking task.

Regarding potential gender differences, the present study adopted an exploratory approach, given the lack of sufficient evidence in the existing literature to support the formulation of specific hypotheses.

## 2 Methods

### 2.1 Participants

Eighty healthy adults (40 females and 40 males) aged between 18 and 26 years (*M*age = 22.62; *SD* = 2.17) volunteered for the study and were mostly recruited from the University campus by word-of-mouth. They were randomly assigned to either the experimental group (i.e., self-selected enjoyable colored environment) or the control group (i.e., neutral gray environment). Participants' physical characteristics and pre-experiment Profile of Mood States (POMS) scores are displayed in Table 1. Exclusion criteria included having a body mass index above 30 (i.e., obesity), being under any medical treatment, or presenting any previous or present health-related disorder or condition that might interfere with their ability to walk normally on a treadmill or to perceive colors (e.g., color blindness). Volunteers were instructed to bring comfortable shoes and sports attire to the laboratory on the testing day. Before the start of the experiment, participants signed an informed consent according to the declaration of Helsinki and the University's code of practice. An institutional review board approval was obtained prior to the start of the study (QU-IRB 1466-EA/21).

**Table 1:** Physical characteristics and pre-experiment profile of mood states (POMS) scores of female and male participants in both the colored and neutral groups.

	**Colored group**			**Neutral group**		
	**Female**	**Male**	**All**	**Female**	**Male**	**All**
Age (years)	22 (1.79)	23 (2.68)	22.50 (2.32)	23 (1.76)	23 (2.31)	22.75 (2.03)
BM (kg)	59 (9.39)	72 (11.58)^**^	65.29 (12.31)	58 (11.55)	74 (11)^**^	65.71 (13.65)
BH (cm)	162 (6.39)	176 (7.49)^**^	168.99 (9.71)	161 (7.15)	175 (9.08)^**^	168.26 (10.58)
**Pre-experiment profile of mood states (POMS)**						
TEN	2.21 (2.20)	2.75 (2.10)	2.49 (2.14)	2.50 (2.75)	3.25 (2.49)	2.89 (2.61)
ANG	2.85 (2.56)	2.58 (2.01)	2.72 (2.28)	2.33 (2.17)	3.05 (1.82)	2.71 (2.00)
DEP	4.80 (3.83)	5.50 (3.68)	5.15 (3.72)	4.65 (4.49)	4.90 (2.90)	4.77 (3.73)
FAT	4.65 (4.57)	4.50 (3.89)	4.57 (4.19)	4.40 (5.50)	4.20 (3.49)	4.30 (4.55)
ERA	11.70 (6.11)	12.45 (5.79)	12.07 (5.88)	11.05 (6.62)	13.10 (7.53)	12.07 (7.08)
VIG	7.95 (2.88)	9.80 (4.07)	8.90 (3.62)	9.85 (4.13)	10.53 (4.94)	10.18 (4.49)
CON	3.45 (3.05)	4.25 (2.43)	3.85 (2.75)	3.06 (3.80)	4.10 (2.67)	3.60 (3.25)
TMD	98.25 (20.66)	97.75 (17.51)	98.00 (18.90)	99.15 (24.21)	95.40 (19.84)	97.27 (21.93)

Values are presented as mean (standard deviation).

^**^Within-group sex differences, *p* < 0.001.

BM, body mass; BH, body height; TEN, tension; ANG, anger; DEP, depression; FAT, fatigue; ERA, esteem-related affect; VIG, vigor; CON, confusion; TMD, total mood disturbance. The POMS clustered mood state scores presented here are the sum of the score of five to seven individual mood states.

### 2.2 Protocol

**Figure 1:**
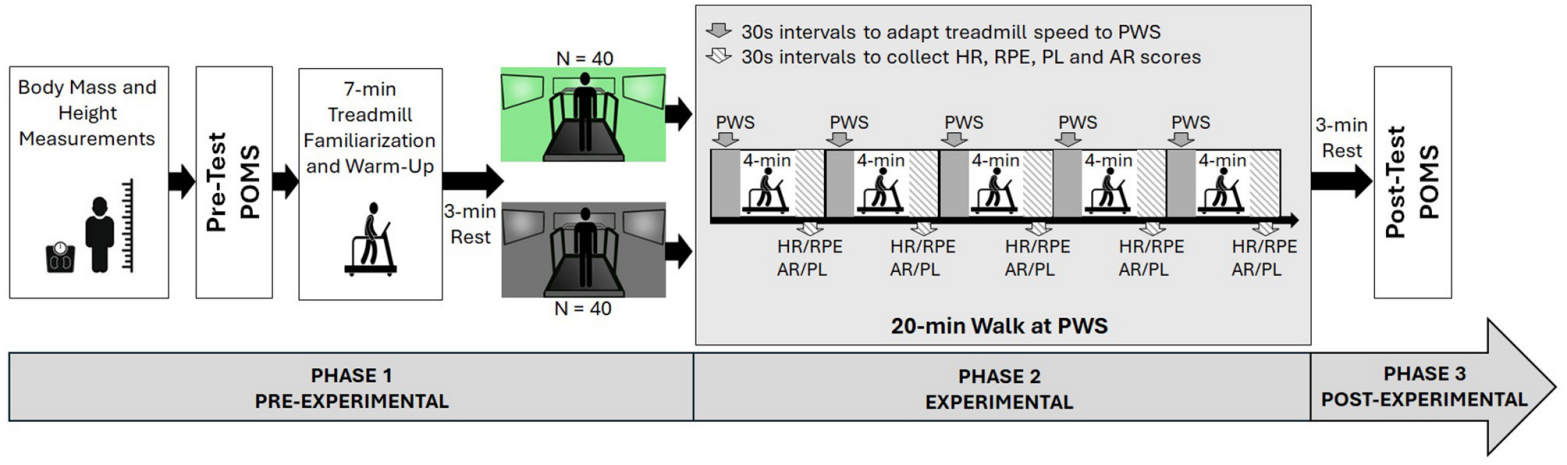
A schematic summary of the study design showing the three phases of the protocol. POMS, profile of mood states questionnaire; PWS, preferred walking speed; HR, heart rate; RPE, rating of perceived exertion; AR, arousal; PL, pleasure.

The protocol consisted of three phases, with a total duration of 60 min, and was performed within a single laboratory visit. All sessions were done under similar environmental conditions of temperature (i.e., 23°C) and relative air humidity (i.e., 60%). Figure 1 summarized the main phases of the protocol.

The first phase started with the assessment of participants' body mass and height, after which a pre-test POMS questionnaire was completed. Standardized instructions on how to report the RPE, pleasure, and arousal scores were given (Schaeffer and Presser, 2003; Terry et al., 2005). For the experimental group (i.e., colored environment), the selection of the most enjoyable color was done by displaying a large variety of colors and fine-tuning the HSV color model until participants reported the color as being their preferred one. Finally, a 7-min walk at different speeds around the preferred one (i.e., higher and lower) was carried out in the neutral gray environment for familiarization with the treadmill and warm-up (Briki and Majed, 2019). This was followed by a 3-min rest, during which a HR monitor was fitted to the participant's chest.

During the second phase (i.e., experimental phase), participants were asked to walk for 20 min on the treadmill at their self-selected, most comfortable speed while their preferred color or a neutral (gray) color was displayed on three large screens at the front and sides of the treadmill, thus creating an immersive visual experience. The 20-min walking task was segmented into five continuous 4-min intervals, each beginning with a 30-s adjustment period during which participants fine-tuned the treadmill speed to maintain their PWS. The adjustments were made by researchers, as participants were blind to the displayed speed. During the final 30 s of each interval, participants verbally reported their ratings of RPE, arousal, and pleasure using scales displayed on the front screen. Meanwhile, HR values were continuously recorded throughout the interval, with the data from this final 30-s period averaged to create the dependent variable for analysis. To reinforce the participant's immersion in the colored environment, the experiment was performed in a dark room, leaving only the sources of color light displayed on the three screens surrounding the treadmill. Moreover, participants did not receive any kind of encouragement or motivation before or during the test.

In the final phase, participants were asked to fill out the post-test POMS questionnaire again after a 3-min resting period.

### 2.3 Materials

Body mass and height were measured using a calibrated scale and portable stadiometer (Seca, Birmingham, UK). The experiment was performed on a medical treadmill with a surface of 60 × 170 cm^2^ and a speed range of 1 to 25 km·h^−1^ with side handrails (Valiant 2 cardio-pulmonary exercise testing, Lode, Netherlands). Three identical 58-inch television screens surrounded the treadmill from the front, left, and right sides (Hisense, Smart Ultra, High-Definition Television, 4K Light-Emitting Diode, China). The three screens displayed the visual properties of the selected color or gray, calibrated using the HSV color model. The latter is considered to be the easiest model in terms of defining colors by individuals' perception (Ibraheem et al., 2012). The colors used were defined according to specific hue, saturation, and luminosity through PowerPoint software (Office 2016, Microsoft). The gray color (neutral condition) was set at a hue of 170, saturation of 0, and luminosity of 128 as done in other studies (Briki et al., 2014, 2015).

HR was continuously measured using a polar chest belt (Polar, Kempele, Finland). RPE were collected using the 15-point Borg scale (Borg, 1973), ranging from 6 (no exertion) to 20 (maximal exertion). Arousal and pleasure were assessed using the 5-point Likert-type scale of the Self-Assessment Manikin (Bradley and Lang, 1994). For arousal and pleasure, respectively, the scale ranged from “I feel excited, nervous, or wide-awake” (scored 5) to “I feel calm, relaxed, or sleepy” (scored 1) and “I feel happy, pleasant, or positive” (scored 5) to “I feel unhappy, unpleasant, or negative” (scored 1), respectively (Nabizadeh Chianeh et al., 2012).

The abbreviated 40-questions POMS questionnaire (Heuchert and McNair, 2012) was used to evaluate mood states (i.e., tension, anger, fatigue, depression, esteem-related effect, vigor, confusion, and total mood disturbance). The answers for the POMS rated on a 5-point Likert-type scale (0 = not at all, 1 = a little, 2 = moderately, 3 = quite a lot, 4 = extremely) to evaluate the intensity of each mood variable (Grove and Prapavessis, 1992).

### 2.4 Data analysis

Characteristics (i.e., HSV model) of individual preference to the most enjoyable color were noted for the experimental group. Data related to PWS, HR, RPE, arousal, and pleasure were collected at each of the five 4-min intervals, while POMS scores were assessed before and after the 20-min walk. The normality of all datasets was verified using Q-Q plots and Shapiro-Wilks tests.

In this study, two power analyses were conducted. Initially, during the study's conception, we recruited 40 participants (20 females and 20 males) based on an *a priori* power analysis performed using G^*^Power software. This analysis was designed to ensure an adequate sample size for detecting moderate effects. Considering the study's 2 (Sex) × 2 (Condition) between-subjects factorial design with repeated measures across five occasions, the expected effect size (*f* = 0.25), significance level (α = 0.05), and statistical power (0.80) were used as parameters. The analysis indicated that a sample size of 32 participants would yield an actual power of 0.83, providing sufficient sensitivity to detect moderate within-between interaction effects.

After data collection and main analyses, trends suggestive of small-to-moderate effects were identified, prompting an expansion of the sample size. This decision was supported by existing literature in color psychology, which consistently indicates that effects in this field are typically small or small-to-moderate (Briki and Majed, 2019; Briki et al., 2015). Adjusting for a new expected effect size (*f* = 0.15), the updated power analysis indicated that a sample size of 80 participants would achieve an actual power of 0.80, providing sufficient sensitivity to detect small-to-moderate effects. Accordingly, an additional 40 participants were recruited, increasing the total sample size to 80.

Two-way analyses of variance (ANOVAs) were used to determine the effect of Condition (i.e., colored and neutral environments) and Sex (i.e., male and female) on the physical characteristics of participants and the pre-experiment POMS clustered scores (Table 1). Mixed ANOVAs examined the within-subject effect of Time (i.e., time intervals: T_1_ [0–4 min], T_2_ [4–8 min], T_3_ [8–12 min], T_4_ [12–16 min], and T_5_ [16–20 min]) and the between-subject effects of Condition (i.e., colored and neutral environments) and Sex (i.e., male and female) on all the dependent variables measured during the 20-min walk (i.e., PWS, HR, RPE, pleasure, arousal). When a trend relative to sex-difference was found (*p* < 0.09), the ANOVA was repeated for male and female groups separately. Further mixed ANOVAs examined the within-subject effect of the 20-min Walking Task (i.e., pre- and post-test) and the between-subject effects of Condition (i.e., colored and neutral environments) and Sex (i.e., male and female) on all POMS scores.

When the assumption of homogeneity was violated, a Huynh-Feldt adjustment was made to the *p* and *F*(degrees of freedom) values (Huynh and Feldt, 1970). Tukey's HSD *post-hoc* pairwise comparisons were used when necessary. All tests were done using SPSS (version 29, IBM SPSS Statistics), with a level of significance set at *p* < 0.05.

## 3 Results

### 3.1 Preliminary results

No significant differences in age, body height, or body mass were detected between the experimental (color) and control (neutral) groups (*p* > 0.05, Table 1). No significant interaction effects were revealed between Condition and Sex on all the variables of the physical characteristics (*p* > 0.05). However, results indicated a significantly higher body height [*F*_(1,76)_ = 63.77, η^2^_p_ = 0.46, *p* < 0.001] and mass [*F*_(1,76)_ = 34.43, η^2^_p_ = 0.31, *p* < 0.001] for males compared to females in the study, while no sex-differences were detected for age [*F*_(1,76)_ = 2.39, η^2^_p_ = 0.03, *p* > 0.05, Table 1]. Moreover, the analyses did not indicate any significant main effect of Sex or Condition on any of the pre-experiment POMS clustered variables namely tension, anger, fatigue, depression, esteem-related affect, vigor, confusion or total mood disturbance (*p* > 0.05).

**Figure 2:**
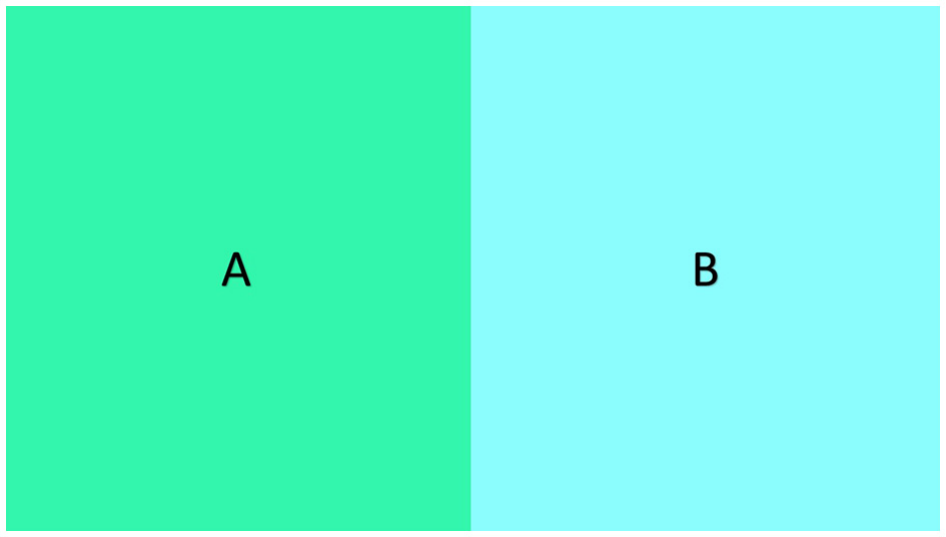
Preferred color represented by the average values of hue, saturation, and luminosity for male **(A)** and female **(B)** groups.

In order to better understand sex differences in the HSV characteristics of color preference, independent *t*-tests were performed. Female and male values for hue and saturation did not differ significantly (*p* > 0.05), while luminosity (i.e., value) was significantly lower for men as compared to women [*t*_(38)_ = 3.94, *p* < 0.001]. The preferred colors for both male and female experimental groups are displayed in Figure 2.

### 3.2 Analyses of variance (ANOVAs)

**Figure 3:**
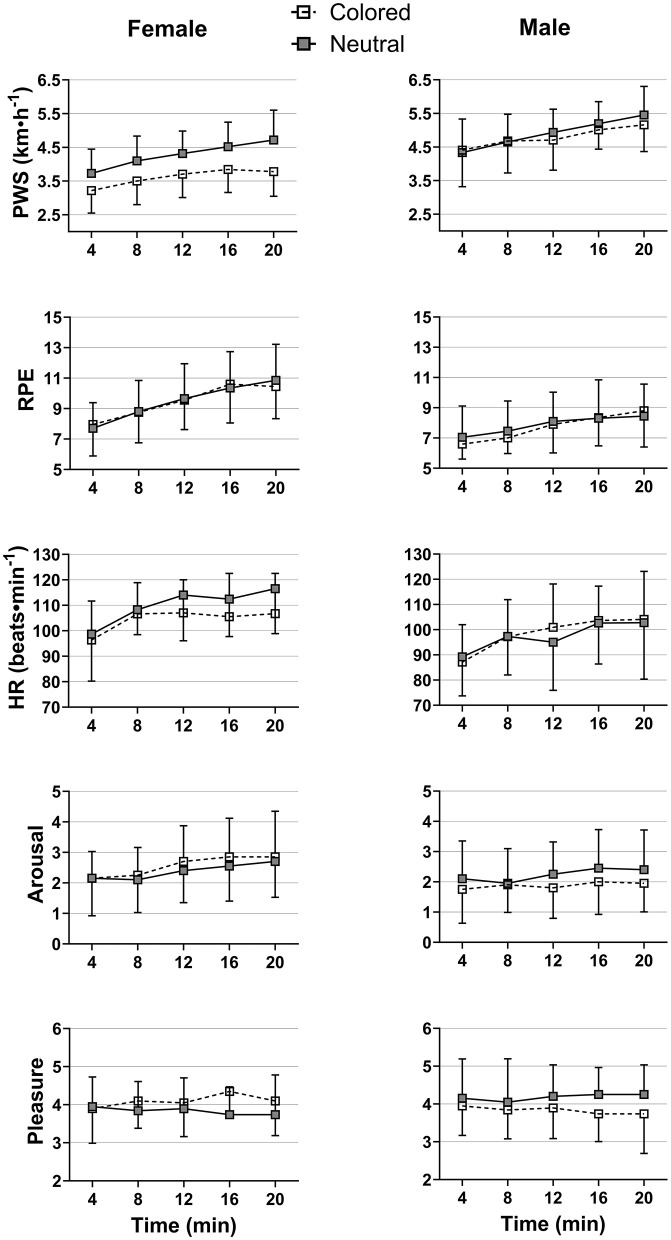
Mean values of main outcomes over the 20-min of walking for both male and female experimental (colored) and control (neutral) groups. Error bars represent the standard errors.

Mixed ANOVAs showed a significant main effect of Condition on PWS [*F*_(1,69)_ = 6.20, η^2^_p_ = 0.08, *p* = 0.015], indicating a significantly lower PWS for participants walking in the self-selected enjoyable colored environment compared to those walking in the gray (neutral) environment (Table 2, Figure 3). A further analysis, conducted separately for male and female groups, revealed that female participants walked slower in the enjoyable colored environment compared to the neutral one [*F*_(1,34)_ = 9.25, η^2^_p_ = 0.21, *p* < 0.005], while males showed no significant differences in PWS between the color conditions [*F*_(1,35)_ = 0.28, η^2^_p_ = 0.008, *p* = 0.60]. No significant main or interaction effects were found on pleasure.

**Table 2:** Results of the mixed ANOVAs regarding the effects of time, sex, condition (and interactions) on preferred walking speed (PWS), rating of perceived exertion (RPE), heart rate (HR), and arousal.

		**PWS**	**RPE**	**HR**	**Arousal**
Time	*df*	1.72, 118.67	2.29, 173.99	3.37, 225.89	2.00, 151.91
	*F*	40.20^***^	72.89^***^	27.89^***^	9.84^***^
	η^2^p	0.37^***^	0.49^***^	0.29^***^	0.11^***^
Sex	*df*	1, 69	1, 76	1, 67	1, 76
	*F*	33.10^***^	15.63^***^	8.89^**^	3.47^#^
	η^2^p	0.32^***^	0.17^***^	0.12^**^	0.04^#^
Condition	*df*	1, 69	1, 76	1, 67	1, 76
	*F*	6.20^*^	0.03	0.15	0.14
	η^2^p	0.08^*^	0.00	0.002	0.002
Time × sex	*df*	1.72, 118.67	2.29, 173.99	3.37, 225.89	1.2, 151.91
	*F*	0.75	4.02^#^	0.78	1.53
	η^2^p	0.01	0.05^#^	0.01	0.02
Condition × sex	*df*	1, 69	1, 76	1, 67	1, 76
	*F*	2.98^#^	0.02	0.14	1.41
	η^2^p	0.04^#^	0.00	0.002	0.02

^#^*p* < 0.09.

^*^p < 0.05.

^**^p < 0.01.

^***^p < 0.001.

*df*, degrees of freedom.

**Figure 4:**
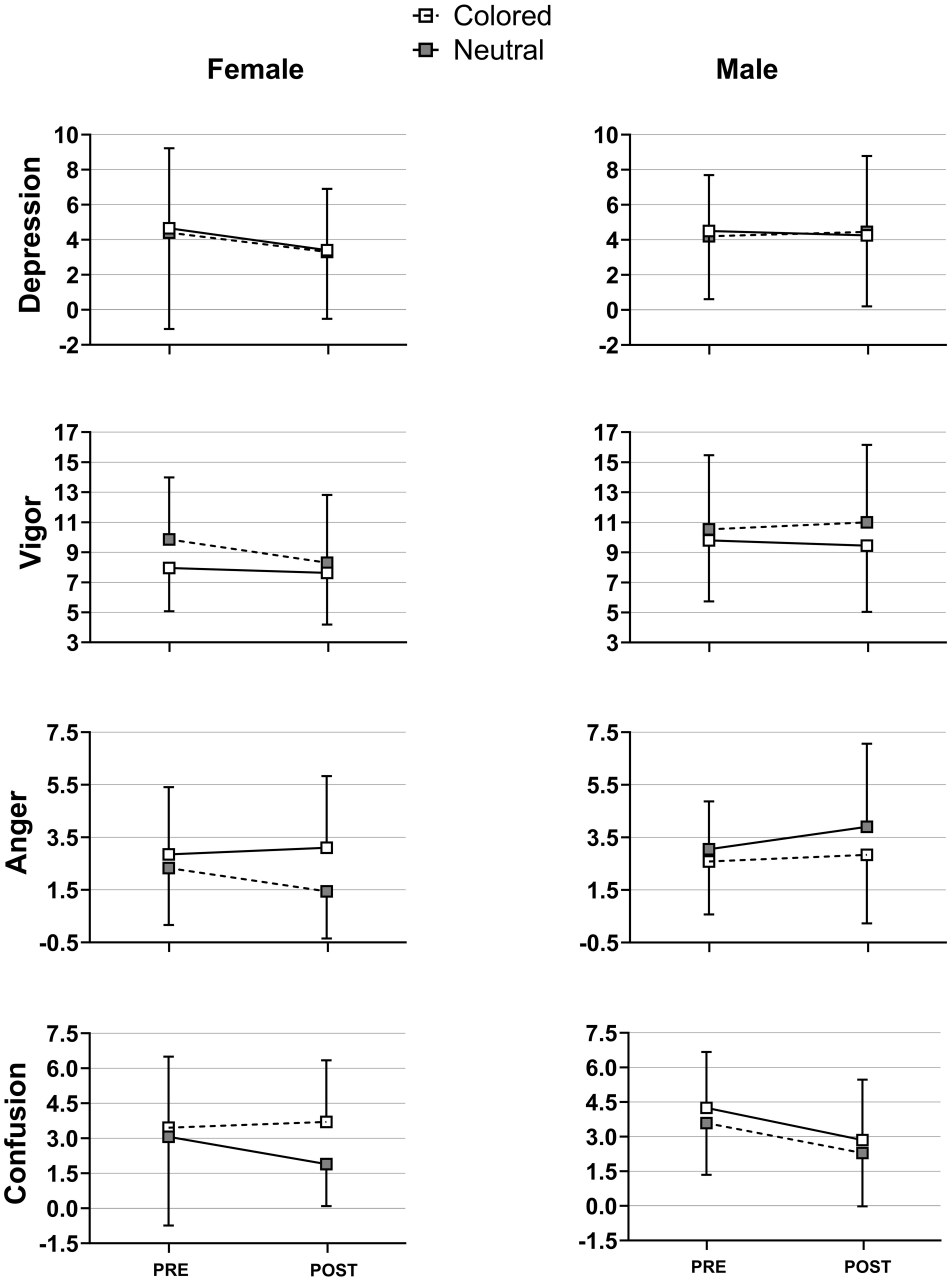
Mean values of main profile of mood states (POMS) outcomes before (pre) and after (post) the 20-min walking for both male and female experimental (colored) and control (neutral) groups. Error bars represent the standard errors.

Results showed a significant main effect of Time on PWS, HR, RPE, and arousal only (Table 2). Figure 2 shows a significant increase in these variables with time intervals. A marginal interaction effect between Time and Sex was detected on RPE showing a higher increase in RPE values for females compared to males (Figure 2). Furthermore, results indicated a significant main effect of Sex on most of the studied variables (Table 2). Although female participants displayed a significantly slower PWS during the trial compared to male participants, they showed significantly higher RPE and HR, as well as marginally higher arousal levels (Figure 4).

Mixed ANOVAs for the POMS revealed a significant main effect of the Walking Task on depression and confusion scores, indicating significantly lower levels of depression and confusion after the 20-min walk as compared to before (Table 3, Figure 3). A significant interaction between Sex and Walking Task was also found on depression. Further analyses done separately for male and female groups revealed that only female participants experienced a significant decrease in depression scores after the 20-min walk [*F*_(1,38)_ = 7.25, η^2^p = 0.16, *p* = 0.01]. Marginal effects are indicated in Table 3 and illustrated in Figure 3.

**Table 3:** Results of the mixed ANOVAs regarding the effects of walking task, sex, condition (and interactions) on profile of mood states: anger, depression, vigor, and confusion.

		**Anger**	**Depression**	**Vigor**	**Confusion**
Walking task	*df*	1, 73	1, 76	1, 74	1, 71
	*F*	0.26	5.01^*^	1.80	14.46^***^
	η^2^p	0.004	0.06^*^	0.02	0.17^***^
Sex	*df*	1, 73	1, 76	1, 74	1, 76
	*F*	1.76	0.21	3.77^#^	0.15
	η^2^p	0.02	0.003	0.05^#^	0.00
Condition	*df*	1, 73	1, 76	1, 74	1, 76
	*F*	0.10	0.02	1.78	2.23
	η^2^p	0.001	0.00	0.02	0.03
Walking task × sex	*df*	1, 73	1, 76	1, 74	1, 71
	*F*	3.52^#^	5.01^*^	2.35	3.50^#^
	η^2^p	0.05^#^	0.06^*^	0.03	0.05^#^
Condition × sex	*df*	1, 32.90	1, 76	1, 74	1, 76
	*F*	3.45^#^	0.00	0.01	0.19
	η^2^p	0.04^#^	0.00	0.00	0.003
Walking task × condition × sex	*df*	1, 73	1, 76	1, 74	1, 71
	*F*	3.41^#^	0.11	2.52	2.57
	η^2^p	0.04^#^	0.00	0.03	0.04

^#^*p* < 0.09.

^*^p < 0.05.

^***^p < 0.001.

*df*, degrees of freedom.

## 4 Discussion

This study explored whether and how individually preferred environmental colors and self-paced walking could influence psychological (i.e., RPE, arousal, pleasure, and mood), physiological (i.e., HR), and behavioral (i.e., PWS) responses during a 20-min walking session, while also exploring potential gender differences in these effects. To our knowledge, existing research on color psychology has largely focused on the inherent effects of predefined colors (e.g., red, green, blue), thus overlooking the potential influence of self-selected, personally preferred colors shaped by cultural and social contexts (Kocher and Sutter, 2008; Lee and Lee, 2021).

Building on the findings of Briki and Majed (2019), this study proposed several hypotheses. First, it was hypothesized that walking in a self-selected, enjoyable colored environment would significantly reduce HR compared to a neutral environment, reflecting the calming effects of immersion in a visually comfortable setting. Second, it was anticipated that RPE, HR, and PWS would progressively increase over time, regardless of the color conditions. Third, self-paced walking was expected to have a positive impact on mood, particularly by increasing vigor from pre- to post-task measures. Finally, regarding gender differences, the study adopted an exploratory approach due to the limited evidence in the existing literature, aiming to identify any emerging patterns or differences—some of which were observed.

### 4.1 Greater female responsiveness to the preferred colored environment

A major finding of this study is that participants walked significantly slower in the colored environment compared to the neutral one, with this effect being more pronounced in females, who substantially reduced their walking speed, whereas males' walking speed remained unaffected (Figure 3). In other words, the overall effect appears to be largely driven by females heightened behavioral sensitivity to the self-selected enjoyable colored environment. This aligns with the findings of Briki and Majed (2019), who reported a significant HR reduction in females walking in a green environment—a color often associated with approach motivation. These results suggest, consistent with Briki and Majed's conclusions, that the decrease in PWS observed in the present study may be attributed to a potential calming effect induced by immersion in a comfortable, enjoyable colored environment.

The hypothesis of a calming effect associated with preferred colors is supported by a study showing that individuals placed in a room lit by their preferred color experienced shorter sleep onset times compared to those in a dark room, suggesting the activation of the parasympathetic nervous system (Lee and Kim, 2017). In a rehabilitation context, this hypothesis is echoed in a study comparing the effects of light-colored rooms to white rooms on psychological wellbeing in 80 patients recovering from hip or knee arthroplasty (Eminovic et al., 2022).

Moreover, contrary to our expectation, the results did not reveal a main effect of the color condition on HR, nor were there any differences observed in RPE, pleasure, or arousal. This prevented us from identifying potential gender-specific differences. A possible explanation for this outcome is that the sample size may have been insufficient to detect small effect sizes, limiting the study's ability to capture subtler effects beyond small-to-moderate ones.

### 4.2 Greater female responsiveness to the walking task

Another major result is that the walking task generally resulted in a significant decrease in levels of depression and confusion, with the reduction in depression being more pronounced in females, while males showed no significant change. First, while this does not directly support our expectation regarding the positive effect of walking on vigor, it is in line with the broader view that walking promotes mood benefits (Briki and Majed, 2019). Furthermore, it reinforces previous findings that self-paced walking significantly enhances enjoyment and affective responses (DaSilva et al., 2009; Ekkekakis and Lind, 2006; Lind et al., 2005). Notably, even a single 10-min bout of walking at a self-selected pace was shown to enhance mood states in both males and females (Edwards and Loprinzi, 2018).

Second, our findings further support the notion of greater psychological responsiveness in females, potentially reflecting underlying gender differences in emotional regulation or sensitivity to environmental and physiological stimuli (Meyers-Levy and Loken, 2015). This heightened responsiveness may make females more attuned to the psychological benefits of physical activity. The results also imply that females may derive greater wellbeing and mental health benefits from *slight* positive stimuli (Meyers-Levy and Loken, 2015), such as those provided by low-intensity exercises like walking, which can act as a subtle yet effective mood enhancer.

Interestingly, although females walked significantly slower during the task compared to males, they revealed higher HR and RPE levels, independent of the experimental conditions (preferred vs. neutral color). Specifically, the maximum RPE reported by males averaged 9, corresponding to a “very light” intensity, whereas for females, it averaged 11, reflecting a “light” intensity. This higher relative intensity in females may have contributed to greater mood-related benefits during the 20-min walk (Noetel et al., 2024). These differences also suggest that disparities in physical conditioning between males and females may have influenced the observed results. This limitation highlights the importance of future studies controlling for participants' physical activity levels and conditioning to better understand these gender-specific responses and their potential implications for exercise interventions.

### 4.3 Gender differences in environmental color preferences

Although secondary, yet equally intriguing, our study revealed gender differences in selected environmental colors. While hue and saturation did not significantly vary between females and males, females appeared to prefer colors with higher luminosity (i.e., value) on the HSV model compared to males. This finding raises questions about how individual HSV characteristics, rather than overall color, may influence motor behaviors. On average, males predominantly selected green hues (i.e., bright mint), while females showed an equal preference for blue and green hues (i.e., cyan), as shown in Figure 2. These results align with Ellis and Ficek's (2001) study, which also reported gender-based differences in color preferences among college students. Their study found that blue and green were the most favored colors, with males showing a stronger preference for blue hues, while females equally favored blue and green (Ellis and Ficek, 2001).

From an evolutionary perspective, in natural settings, green and blue are commonly associated with the sky, nature, water (e.g., seas or oceans), and food availability (Wilson, 1984). These colors are thought to reflect resources that historically offered advantages for survival, fertility, and social cohesion (Palmer and Schloss, 2010). This understanding has potential applications in exercise psychology, where the choice of an exercise environment could influence individuals' lived experiences and motivation levels. For instance, walking along the sea may appeal to those who prefer blue hues, while walking in a park or forest might attract individuals with a preference for green hues. However, due to rapid urbanization worldwide, many outdoor activities have shifted to indoor settings (Gladwell et al., 2013). In such cases, adapting exercise environments with enjoyable colors can provide psychological benefits for individuals who prefer or are limited to indoor exercise (e.g., gyms, clinical settings, or during adverse environmental conditions such as heat, humidity, or high pollution).

The ecological valence theory suggests that people develop color preferences based on positive experiences (Palmer and Schloss, 2010). Moreover, culture has been identified as an essential factor contributing to gender differences in color preferences (Davis et al., 2021). While stereotypes often link certain colors to boys or girls (Del Giudice, 2012, 2017), research indicates that these associations are cultural rather than innate (Alexander, 2003; Davis et al., 2021). This is further supported by studies showing no gender differences in color preferences among infants aged 3 to 24 months (Franklin et al., 2010).

However, the fact that females selected colors with higher luminosity may be rooted in potential genetic differences. The genes encoding most photopigments are located in small arrays on the X chromosome (Nathans et al., 1986), and evidence suggested that females would be generally better at identifying shades, brightness, and light characteristics of colors compared to males (Jaint et al., 2010; Mäntyjärvi and Tuppurainen, 1995). This genetic difference may explain why females would be more likely to select colors with higher luminosity, as they may perceive and differentiate subtle variations in light and color more effectively.

### 4.4 Self-paced walking: a pleasure-driven dynamic?

Consistent with our expectations, the findings showed a significant increase in RPE, arousal, HR, and PWS over time, irrespective of the color condition. These results are consistent with previous studies showing similar increases in RPE, HR, and preferred intensity or speed during relatively short durations of low-intensity exercise (Briki and Majed, 2019; Smith et al., 2015). Evidence suggests that during such exercise, individuals tend to gradually approach their ventilatory threshold (Smith et al., 2015). Conversely, it was proposed that people typically select a walking speed that optimizes mechanical efficiency and minimizes metabolic costs, potentially reducing carbohydrate consumption and aiding in fatigue prevention (Willis et al., 2005). However, in our study, participants progressively increased their PWS over time, regardless of color conditions and sex, despite simultaneous increases in psychophysiological responses (HR, RPE, arousal). This suggests that factors beyond metabolic or physiological optimization may play a significant role in influencing participants' inclination to walk faster, potentially driven by complex, largely unconscious processes.

Interestingly, pleasure levels remained stable throughout the walking task, with an average score of 4 (Figure 3), reflecting feelings described as “moderately happy, pleasant, or positive” (Bradley and Lang, 1994). In contrast, arousal displayed a steady increase over the 20-min period (Figure 3). This pattern suggests a dynamic interplay in which participants may instinctively adjust their pace and effort to sustain a consistently pleasurable experience. Thus, our findings suggest the notion that self-paced walking would be intrinsically governed by a pleasure-driven dynamic. This aligns with the concept of “dynamical system,” defined as “a set of interconnected elements that influence each other to achieve a common or coordinated state,” where stability is maintained by an influential variable, called “attractor,” which enables the system to resist external disturbances (Vallacher et al., 2023, p. 24). From this perspective, engaging in self-paced walking can be viewed as a dynamical system in which physiological, biomechanical, and psychological factors interact in a complex manner to produce a coordinated state—walking faster, accompanied by increased cardiorespiratory activity, arousal, and RPE—driven by the attractor of moderate pleasure.

### 4.5 Limitations

Although this study had the advantage of exploring the potential effects of perceiving self-selected enjoyable colored environments (rather than a pre-defined set color), some limitations should be considered. Since this study only included participants living in Qatar and aged 18–26 years, the findings may not be generalizable to other age groups or populations. Additionally, the controlled laboratory setting might not fully reflect the psychophysiological response to colors in real-world conditions. Furthermore, this study did not assess the participants cardiorespiratory fitness or physical activity levels and did control for confounding variables related to diet, sleep, previous exercise, and physical conditioning levels. Future studies could consider graded exercise tests for assessing cardiorespiratory fitness levels to know precisely at what relative intensities participants were walking. This would better address physiological responses and provide further insights into the determinants of self-selected exercise intensities and behaviors, in addition to the effect of colors. Future researchers may also consider examining the long-term psychological, physiological and behavioral impact of perceiving the enjoyable colored environments during exercise. Future research should consider larger sample sizes to improve statistical power and better identify small but meaningful effects. Finally, such studies would deepen our understanding of how environmental factors impact physical activity and overall wellbeing, offering new avenues for personalized approaches in exercise psychology.

### 4.6 Practical implications

The results of this study suggest that adapting indoor environments with enjoyable colors may be particularly effective for low-impact or low-intensity activities that promote relaxation, such as yoga, walking, or physical rehabilitation, especially for females. These findings highlight the potential for incorporating preferred colors in rehabilitation settings to enhance motivation and foster positive psychological adaptations in female patients. For example, walls, furniture, and even informational materials such as brochures or invitations could be designed using colors that align with patients' preferences, creating an environment that promotes a sense of comfort and engagement. This approach may be particularly beneficial in clinical rehabilitation programs, where sustained participation and psychological wellbeing are critical to achieving optimal outcomes.

## Data Availability

The original contributions presented in the study are included in the article/supplementary material, further inquiries can be directed to the corresponding author.
